# Expression profiles of long non-coding RNAs ROCR, CFLAR-AS1, ST7-AS1, and ST7-AS2 in diffuse large B-cell lymphoma: a case-control study focusing on GCB and non-GCB subtypes

**DOI:** 10.1186/s12920-026-02452-0

**Published:** 2026-08-10

**Authors:** Ali Rajabi, Maryam Ghaffari Laleh, Reza Safaralizadeh, Asma Rajabi, Samaneh Tayefeh-Gholami, Ali Saber

**Affiliations:** 1 Department of Animal Biology, Faculty of Natural Science, University of Tabriz, Tabriz, Iran; 2 Department of Medical Biology, Faculty of Medicine, Ege University, Izmir, Turkey; 3 Hematology and Oncology Research Center, Tabriz University of Medical Sciences, Tabriz, Iran; 4 Dr. Saber Medical Genetics Laboratory, Rasht, Gilan Iran

**Keywords:** Large B-cell Lymphoma (DLBCL), Germinal Center B-cell (GCB), Non-Germinal Center (non-GCB), Activated B-cell (ABC), Long Non-coding RNA (lncRNA)

## Abstract

**Background:**

Diffuse Large B-cell lymphoma (DLBCL) is subdivided into the Germinal Center B-cell (GCB) and non-Germinal Center B-cell (non-GCB)/Activated B-cell (ABC) subtypes, with significant clinical, phenotypic, and genetic variability. Investigating DLBCL’s molecular mechanisms and developing tailored treatment strategies at the molecular level could improve diagnostic precision and treatment outcomes. Advances in sequencing technologies have enabled the identification of numerous lncRNAs with abnormal expression in DLBCL. This study aimed to identify lncRNAs with diagnostic potential in DLBCL using bioinformatics, confirmed through laboratory experiments.

**Method:**

The gene expression dataset for GSE207388 was obtained from the Gene Expression Omnibus database. Significance analysis of the RNA sequencing algorithm was used to identify differentially expressed genes (DEGs), followed by GO and KEGG enrichment analyses of co-expressed genes. Lymph node tissue specimens were collected from 261 DLBCL patients and 123 controls at two hospitals in Iran between 2023 and 2024. Quantitative real-time PCR was performed to evaluate gene expression, and ROC curve analysis was conducted to assess the diagnostic potential of the selected lncRNAs.

**Results & conclusion:**

The dataset included the expression profiles of 19,305 genes, of which 299 were selected using the cut-off criteria of fold change > 2 and p ≤ 0.001. Based on GO and KEGG enrichment analyses, four key lncRNAs—ROCR, CFLAR-AS1, ST7-AS1, and ST7-AS2—were selected for further analysis. Elevated expression levels of ROCR, CFLAR-AS1, ST7-AS1, and ST7-AS2 were observed when comparing DLBCL versus control groups and non-GCB versus GCB subtypes. Additionally, ROC curve analysis revealed the strong diagnostic potential of these lncRNAs, particularly ST7-AS2. These findings significantly enhance our understanding of the molecular mechanisms underlying DLBCL, potentially leading to more precise therapeutic strategies.

**Supplementary Information:**

The online version contains supplementary material available at https://doi.org/10.1186/s12920-026-02452-0.

## Background

Lymphoma, a malignant tumor originating in the lymphatic and hematopoietic systems [1], is characterized by main manifestations of hepatosplenomegaly, painless lymphadenopathy, and systemic symptoms such as fever and weakness. It is classified into two main types: Hodgkin’s Lymphoma (HL) and Non-Hodgkin’s Lymphoma (NHL) [2]. NHL is further divided based on the origin of the cells, including B, T, and the natural killer cell lymphomas. Diffuse Large B-cell Lymphoma (DLBCL) is the most prevalent subtype, accounting for 40% of all NHL diagnoses in Western countries [3]. DLBCL, a highly aggressive malignancy, primarily involves lesions in the lymph nodes, but some are also detected in the central nervous system, gastrointestinal tract, and bone. DLBCL is subdivided into the Germinal Center B-cell (GCB) subtype and the non-Germinal Center/Activated B-cell (non-GCB/ABC) subtype, based on the differentiation in immunohistochemistry and gene-expression profiles [4]. The non-GCB/ABC subtype is associated with mutations in genes involved in NF-κB signaling, B-cell receptor (BCR) signaling, and toll-like receptor signaling pathways [5], while mutations and translocations in BCL2, BCL6, MYC, and EZH2 genes are frequently observed in the GCB subtype [6]. Consequently, DLBCL patients exhibit heterogeneous clinical responses to standard therapies, ranging from recovery to treatment failure, refractory disease, or even death [7]. Research has shown that non-GCB patients have poorer survival and prognosis compared to GCB patients, with a weaker response to common therapies such as Rituximab combined with the CHOP regimen [8, 9]. However, treatments targeting the NF-κB pathway, such as Bortezomib and Lenalidomide, have demonstrated better clinical outcomes in the non-GCB group [10]. Recent advances in innovative treatment plans, such as stem cell transplantation and combined chemotherapy-immunotherapy, have significantly improved the overall prognosis for DLBCL patients. Nevertheless, 30–40% of DLBCL patients develop treatment resistance or do not respond to current therapies. Therefore, from a clinical perspective, investigating the molecular mechanisms of DLBCL and developing tailored treatment strategies at the molecular level are essential to improve diagnostic precision and treatment outcomes [7, 11].

Noncoding RNAs comprise more than half of the human genome that is actively transcribed. RNAs without coding functions that have a length exceeding 200 nucleotides are referred to as long noncoding RNAs (lncRNAs). Due to splicing, polyadenylation, and 5ʹ capping, lncRNAs share structural similarities with mRNA [11]. Recent research has revealed that lncRNAs are aberrantly expressed in several cancer types, including breast cancer, non-small-cell lung cancer, gastric cancer, hepatocellular carcinoma, prostate cancer, and pancreatic cancer [11, 12]. It has been shown that lncRNAs, as epigenetic regulators, influence tumor development by controlling the expression of genes involved in the immune system, pluripotency, cell cycle, and cell survival [13]. In a transcriptome sequencing study, 2,632 novel lncRNAs were identified in DLBCL tumors, in which two-thirds of these lncRNAs are not expressed in normal B-cells, while one-third are expressed differently in DLBCL subtypes [14]. Peng et al. discovered a higher expression of lncRNA LUNAR1 in DLBCL cell lines and tumor tissues, and knockdown of LUNAR1 resulted in increased expression of p21 (a mediator of cell cycle arrest) and downregulation of the transcription factors E2F1 and cyclin D1, which prevented cell growth [15]. In another similar experiment, Fu et al. suggested a functional role for lncRNA SBF2-AS1 as an accelerator in DLBCL cell growth and tumorigenesis via miR-494-3p/FGFR2 axis [11]. Additionally, the research showed that the expression levels of SNHG8, FIRRE, SNHG14, and MALAT1 lncRNAs were upregulated in DLBCL [13, 16, 17]. All these studies suggest the potential role of lncRNAs as biomarkers for diseases diagnosis and prognosis [13].

The advancement of high-throughput transcriptome sequencing techniques, including RNA-seq, has opened new opportunities for characterizing gene expression patterns across different cell types and conditions [18]. Several studies have explored potential prognostic biomarkers and new potential druggable therapeutic targets for DLBCL at the genomic level [19–22]. In the current study, data mining from the Gene Expression Omnibus (GEO) [19] identified four unique markers (ROCR, CFLAR-AS1, ST7-AS1, and ST7-AS2) based on their significantly different expression between GCB and non-GCB subtypes.

Among these candidates, CFLAR-AS1 (Caspase 8 and Fas-associated via death domain like apoptosis regulator antisense RNA 1) is located on Chromosome 2 [23]. Numerous isoforms of the gene CFLAR, such as those that cause apoptosis or inhibit the death-receptor-induced apoptosis pathway, stop CASP8 activation [24]. Elevated expression of CFLAR-AS1 in patients with Esophageal Squamous Cell Carcinoma (ESCC) suggests its potential as a biomarker for predicting the early occurrence of ESCC [25, 26].

ST7 antisense RNA1 (ST7-AS1) and ST7 antisense RNA2 (ST7-AS2) have been identified in chromosome 7q31.2. ST7-AS1 with Copy Number Variations may induce gene failure in tumors downstream of cancer; it is also highly associated with cell migration, apoptosis, and proliferation. Moreover, ST7-AS1 is a tumor suppressor or oncogene in several tumor types [27, 28].

Recent research has revealed that lncRNA ROCR (Regulator of Chondrogenesis RNA), also known as LINC02095, promotes the oncogene SOX9 and causes cancer cell proliferation [29]. The ROCR is expressed from a locus 94 kb upstream of SOX9 in chromosome 17. It has been indicated that the ROCR locus may play a role in the in vivo control of SOX9 [30].

In the present study, after screening the RNA-Seq expression profiles of DLBCL cell lines extracted from the GEO database, we assessed the expression levels of identified lncRNAs in an Iranian population. We found significantly higher expression levels of CFLAR-AS1, ST7-AS1, and ST7-AS2 in the non-GCB subtype compared with the GCB subtype, whereas ROCR expression was significantly higher in the GCB subtype than in the non-GCB subtype.

## Materials & methods

### Datasets & data retrieval

Gene Expression Omnibus (GEO) database was searched for retrieving Gene expression datasets, resulting in the selection of GSE207388 (https://www.ncbi.nlm.nih.gov/geo/query/acc.cgi?acc=GSE207388). “B-cell lymphoma” was used as a keyword, with the following inclusion criteria: Organism: Homo sapiens; Study type: Expression profiling by array, Expression profiling by high throughput sequencing, and Non-coding RNA profiling by high throughput sequencing. All the CEL raw files were downloaded using the R language analysis tool.

### GO and KEGG enrichment analysis

To investigate the potential biological functions of the selected lncRNAs, Gene Ontology (GO) biological process and Kyoto Encyclopedia of Genes and Genomes (KEGG) pathway enrichment analyses were performed based on their co-expressed protein-coding genes, as lncRNAs are not directly annotated in these databases. Pearson correlation analysis was conducted between each lncRNA and all protein-coding genes across the transcriptomic dataset, and genes with a strong correlation (|r| > 0.6) were selected for enrichment analysis. GO and KEGG enrichment analyses were performed using the clusterProfiler R package with the org.Hs.eg.db annotation database for GO analysis. The Benjamini–Hochberg method was applied to adjust for multiple testing, and an adjusted P value < 0.05 was considered statistically significant.

### Recruitment of participants & sample collection

The investigation was conducted at Bu Ali Hospital in Tehran and Shahid Ghazi Hospital in Tabriz between January 2023 and December 2024. Tissue specimens from lymph nodes were collected from 261 DLBCL patients, comprising 101 GCB and 160 non-GCB subtypes. The control group consisted of 123 age- and sex-matched individuals who underwent diagnostic biopsies for suspected lymphoproliferative conditions, but were histologically diagnosed with benign reactive lymphoid hyperplasia. These non-malignant specimens served as the baseline cohort. The diagnosis and subtypes of DLBCL were reviewed and classified according to the 2016 WHO classification revision of lymphoid neoplasms by a hematopathologist [31]. Reviewing the medical records of each patient allowed for the collection of clinical, laboratory, and pathological data. Table 1 provides an overview of the patients’ clinicopathological characteristics, including gender, age, weight, B symptoms, serum LDH levels, Ann Arbor stage, and Eastern Cooperative Oncology Group (ECOG) performance status. The ethics committee of University of Tabriz approved the study protocol, and each participant completed a written informed consent form to participate in the research (Approval number: IR.TABRIZU.REC.1404.097).

**Table 1:** Clinicopathologic characteristics of GCB and non-GCB patients

characteristics	GCB *N* = 101	Non-GCB *N* = 160	*P*-value	OR (95% CI)
Age (in years)				
≤ 60	42	69	0.806	0.939 (0.567–1.555)
> 60	59	91		
Gender				
Female	58	68	0.019*	0.548 (0.331–0.907)
Male	43	92		
Ann Arbor stages				
I/II	44	55	0.136	1.474 (0.884–2.457)
III/IV	57	105		
B symptoms				
Absent	46	77	0.684	1.109 (0.673–1.828)
Present	55	83		
LDH serum levels				
Normal	48	84	0.434	0.819 (0.498–1.349)
High	53	76		
Bulk				
≥ 2 cm	59	100	0.51	0.843 (0.507–1.402)
< 2 cm	42	60		
ECOG score				
0–1	74	103	0.134	1.517 (0.878–2.620)
≥ 2	27	57		

### Quantitative real-time Reverse Transcription Polymerase Chain Reaction (qRT-PCR)

TRIzol reagent (Invitrogen, Massachusetts, USA) was used to extract total RNA from tissue samples following standard procedures. DNase I was then applied to the RNA samples to prevent genomic DNA contamination. The quantity and quality of the RNA samples were assessed using a NanoDrop and 1% agarose gel electrophoresis. Total RNA was reverse-transcribed to create complementary DNA (cDNA) for RT-PCR using a TaKaRa kit. The reaction mixture was incubated for five seconds at 85 °C and for sixty minutes at 37 °C. All cDNA and RNA samples were stored at -80 °C. The primers for QRT-PCR studies are listed in Table 2.

**Table 2:** Primer sequences to amplify four key genes and the internal control gene

Gene name	Primer sequence	Primer length	Product length
ROCR	F: TACAGTCAAATGCGTGCTGC R: TAGCGGTCTGCGAAACCATC	20 bp	166 bp
CFLAR-AS1	F: CTGCACTCCTCCCAAGTGTT R: CCCAGAATCCTGCCTTGGTT	20 bp	170 bp
ST7-AS1	F: TCGCGTTCTGGGTCCATTTA R: CTGCCTGAGGCGTTTCCTTC	20 bp	86 bp
ST7-AS2	F: GGTGATGGAAGGCAAGGTCA R: GTGGTGGGTATGACCAGTGT	20 bp	134 bp
β-actin	F: AGAGCTACGAGCTGCCTGAC R: AGCACTGTGTTGGCGTACAG	20 bp	184 bp

We used a LightCycler^®^ 96 Real-Time PCR system (Roche Molecular Systems, Inc., Pleasanton, USA) to measure the expression levels of the ROCR, CFLAR-AS1, ST7-AS1, and ST7-AS2 by amplifying the cDNA and assessing it with SYBR Green Master Mix (Amplicon, Odense, Denmark). The β-actin housekeeping gene was used as an internal control to standardize the expression. All amplifications were carried out in triplicate to ensure technical reproducibility, and a melting curve analysis was performed immediately following cycle completion to verify product specificity. The calculated cycle threshold (C_t_) values were used to determine the relative expression of the target lncRNAs using the 2^−ΔCt^ method, where ΔC_t_ represents the difference between the target lncRNAs and the β-actin reference gene.

### Statistical analysis

Statistical analyses were carried out using the R statistical package (version 3.4.2), and differentially expressed genes (DEGs) were visualized using the R packages pheatmap and ggplot2. Statistical significance was determined using the Kruskal–Wallis and log-rank tests. Results for each group were presented as mean ± SD. The student’s t-test examined differences between the two groups. In addition, SPSS software (version 27) was used to conduct a two-way analysis of variance (ANOVA) test to evaluate differences between several groups. GraphPad Prism software (version 9) was utilized to compare areas under curves (AUC) to quantify diagnostic performance through time-dependent Receiver Operating Characteristic (ROC) curve analysis. Two-sided p-values are used, with *p* < 0.05 being regarded as significant.

## Results

### GEO dataset analysis and differential expression in DLBCL

**Fig. 1:**
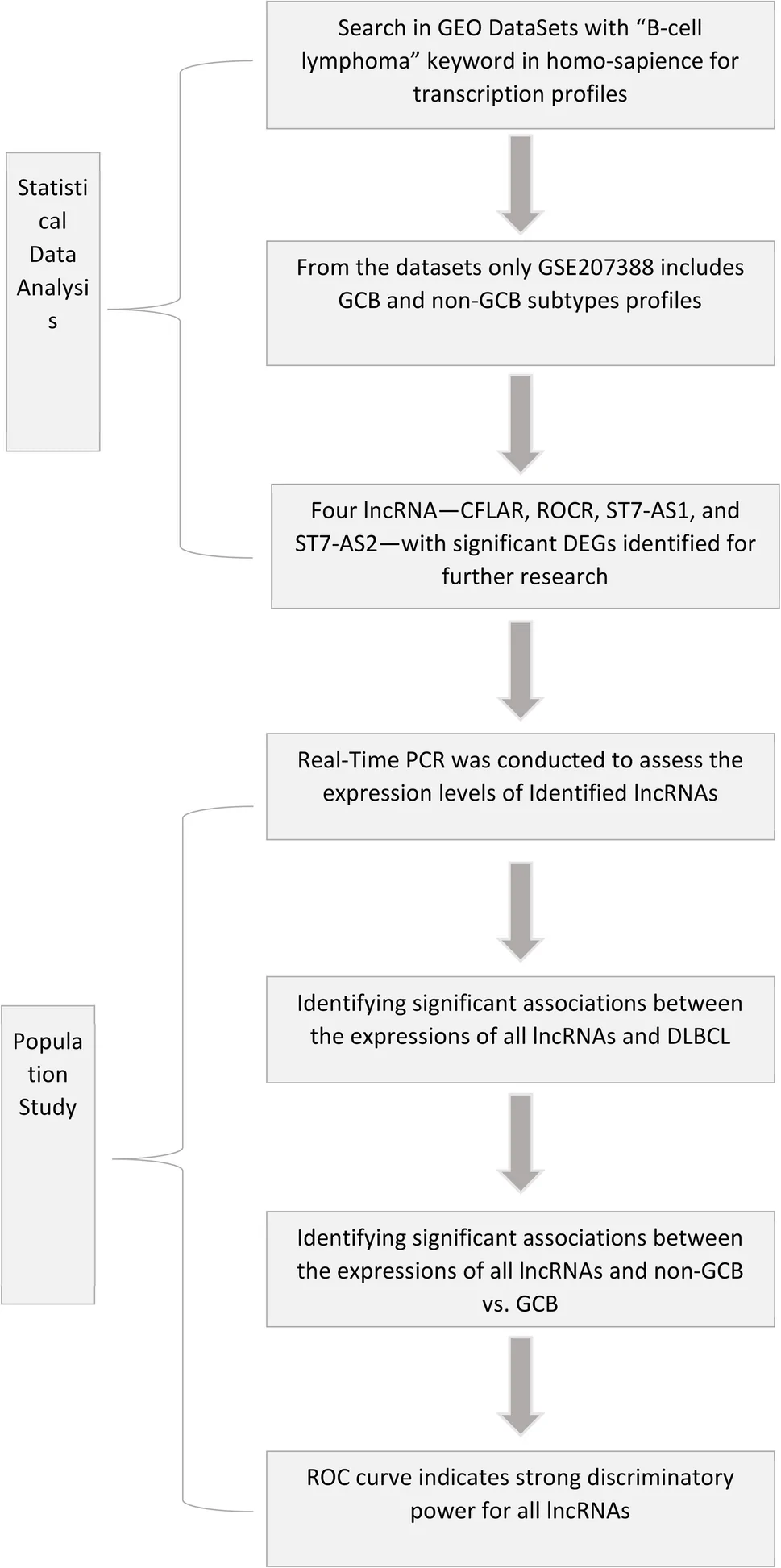
Flow chart of the research design

**Fig. 2:**
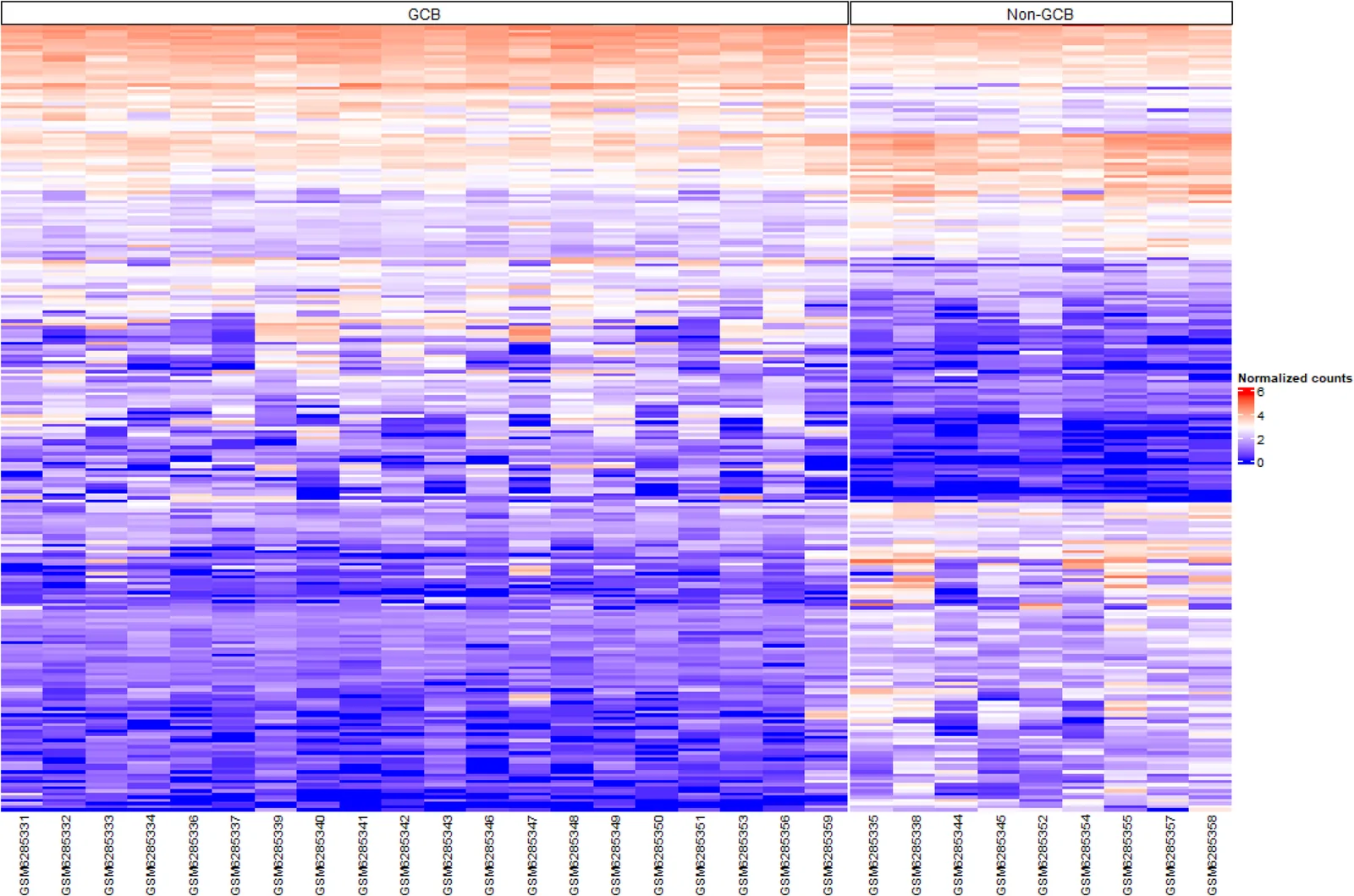
Heatmap of DEGs. The columns represent individual samples from the GCB and non-GCB subtypes; the rows indicate genes showing significant differences in expression between these subtypes

**Fig. 3:**
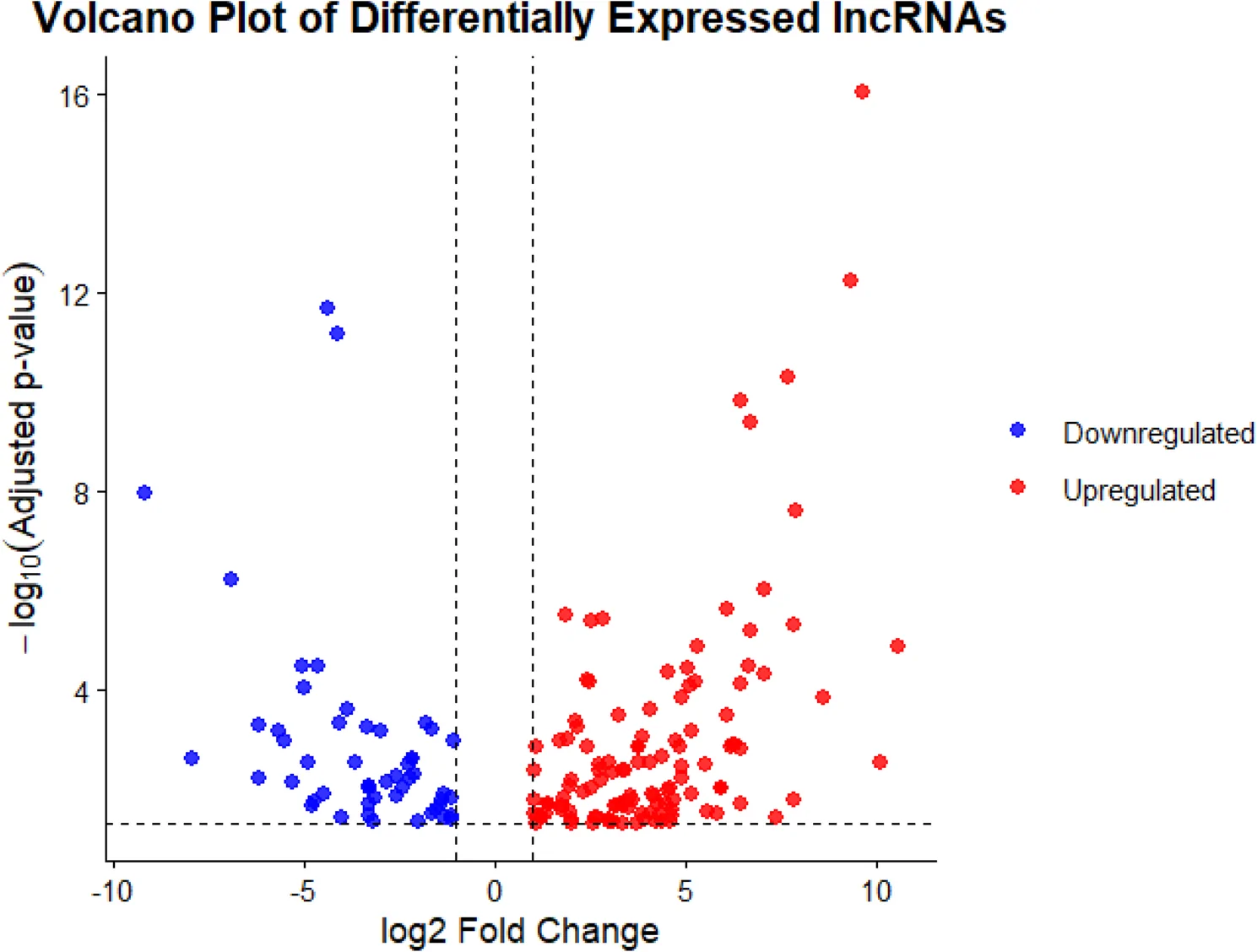
Volcano plot of differentially expressed lncRNAs in DLBCL. Red and blue dots represent significantly upregulated and downregulated lncRNAs, respectively, in the non-GCB subtype compared to the GCB subtype (|log_2_ FC| > 1, adjusted *p* < 0.05)

To determine the DLBCL diagnostic signature, we carried out a multistep study. The flow chart for the workflow of this study is shown in Fig. 1. After filtering the GEO database, we selected GSE207388 deep sequencing datasets with differential expression. The GSE207388 dataset included 29 RNA-Seq samples from DLBCL cell lines (20 GCB and 9 non-GCB) and gene expression profiles of a total of 19,305 genes, including 1,742 with dysregulated expression. Differential expression analysis was performed using DESeq2, and 175 significantly dysregulated lncRNAs were identified using the criteria padj < 0.05 and |log2FC| > 1. The heatmap and volcano plot display the DEGs between the GCB group and non-GCB group (Figs. 2 and 3). Four key lncRNAs were chosen based on their data availability for further expression analysis.

### Functional enrichment analysis of lncRNAs co-expressed genes

To explore the potential biological roles of the selected lncRNAs, we analyzed their co-expressed protein-coding genes, as lncRNAs lack direct annotations in the GO and KEGG databases. Based on a Pearson correlation analysis across 30,842 transcripts, genes with strong correlations (|r| > 0.6) were selected for functional enrichment profiling.

For CFLAR-AS1, 912 co-expressed genes were identified. GO analysis revealed a strong enrichment in the adaptive immune response based on somatic recombination of immune receptors built from immunoglobulin superfamily domains (Adjusted *p* = 5.84 × 10^− 7^). Other enriched processes included regulation of T-cell activation and Th17 cell differentiation. Meanwhile, KEGG pathway analysis identified significant enrichment of immune- and cytokine-related signaling pathways, significantly led by the JAK–STAT signaling pathway and cytokine–cytokine receptor interaction (Adjusted *p* = 4.91 × 10^− 4^ for both), followed by viral protein interaction with cytokine and cytokine receptor, TNF signaling pathway, Toll-like receptor pathway, and hematopoietic cell lineage.

For ST7-AS1, we identified 486 co-expressed genes. GO enrichment analysis identified Th17-type immune response and Th17 cell differentiation as the most significantly enriched biological processes (Adjusted *p* = 9.66 × 10⁻⁴), followed by adaptive immune response based on somatic recombination of immune receptors and T-cell differentiation involved in immune response. KEGG analysis further supported these findings, showing significant enrichment in the Th17 cell differentiation and osteoclast differentiation pathways (Adjusted *p* = 2.85 × 10^− 2^ for both).

For ST7-AS2, 520 co-expressed genes were identified. GO enrichment analysis showed highly significant enrichment of Th17 cell differentiation (Adjusted *p* = 4.09 × 10⁻⁹; fold enrichment = 22.01), together with Th17-type immune response, adaptive immune response, T-cell differentiation involved in immune response, CD4⁺ αβ T-cell differentiation, and positive regulation of leukocyte cell-cell adhesion. This closely aligned with the KEGG pathway analysis, which identified Th17 cell differentiation as a major enriched pathway (Adjusted *p* = 9.18 × 10^− 4^). Additional enriched pathways included the JAK–STAT, TNF, and NF-κB signaling pathways, the PD-L1/PD-1 checkpoint pathway in cancer, osteoclast differentiation, proteoglycans in cancer, and lipid and atherosclerosis.

For ROCR, no significantly enriched GO terms or KEGG pathways were identified among its co-expressed genes using the predefined significance threshold.

Functional enrichment analysis showed that the majority of co-expressed genes were enriched in immune-related biological processes. Th17 cell differentiation and adaptive immune responses were consistently enriched among CFLAR-AS1, ST7-AS1, and ST7-AS2, suggesting shared involvement in immune regulation.

### Association of lncRNAs expression with DLBCL and clinical variables

Tissue specimens from 261 DLBCL patients, including 101 GCB and 160 non-GCB subtypes, (mean ± SD: 63.85 ± 11.97) and 123 healthy controls (mean ± SD: 63.93 ± 11.88) were included in this study. No statistical differences were observed in the age or gender ratio between patients and controls (Table 1).

**Fig. 4:**
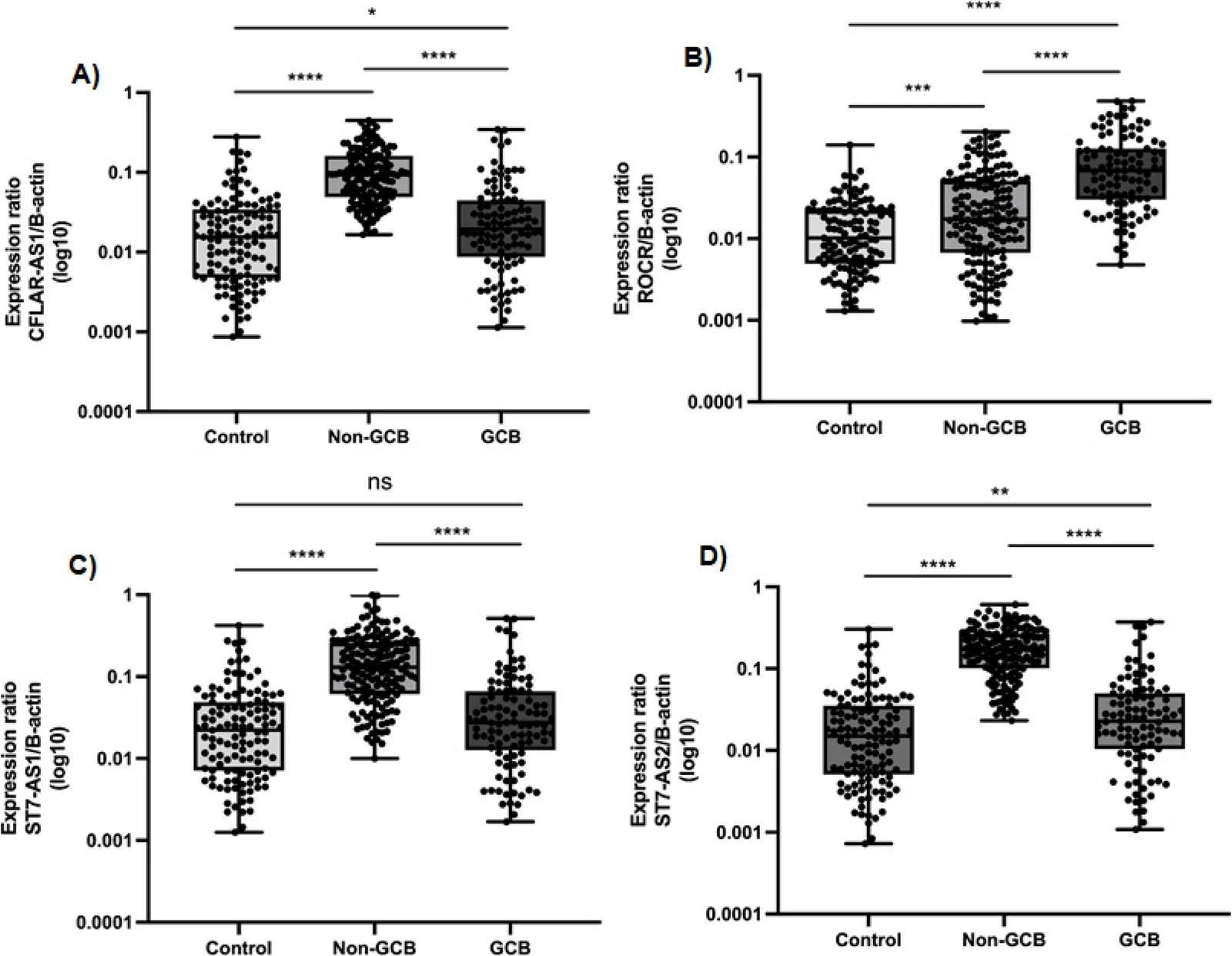
Gene expression analysis of four key genes. **A**) CFLAR-AS1; **B**) ROCR; **C**) ST7-AS1; **D**) ST7-AS2. Data are presented as mean ± SD. Significance levels are indicated as ns: *P* > 0.05; *: *P* ≤ 0.05; **: *P* ≤ 0.01; ***: *P* ≤ 0.001; ****: *P* ≤ 0.0001

Gene expression analysis of four lncRNAs is shown in Fig. 4. The expressions of ROCR, CFLAR-AS1, ST7-AS1, and ST7-AS2 were significantly upregulated in DLBCL samples compared to controls (all with *P* < 0.0001). Considering the subtypes, the expression levels of ROCR, CFLAR-AS1, and ST7-AS2 were significantly higher in both the GCB and non-GCB subtypes compared to the control group. However, ST7-AS1 expression was significantly increased only in the non-GCB subtype compared to the control group. Additionally, significant differences in the expression of all four lncRNAs (ROCR, CFLAR-AS1, ST7-AS1, and ST7-AS2) were observed between the non-GCB and GCB groups (all with *P* < 0.0001). CFLAR-AS1, ST7-AS1, and ST7-AS2 showed higher expression levels in the non-GCB group than in the GCB group, whereas ROCR expression was lower in the non-GCB group than in the GCB group.

We analyzed the correlation between gene expression and clinical characteristics of GCB and non-GCB subtypes (Table 3). The only significant association was found for ROCR with the characteristic of B symptoms (*p* = 0.04).

**Table 3:** Expression levels (mean ± SD) of CFLAR-AS1, ROCR, ST7-AS1, and ST7-AS2 according to clinicopathological characteristics in GCB and non-GCB DLBCL patients

Genes Characteristics	CFLAR-AS1			ROCR			ST7-AS1			ST7-AS2		
	GCB	Non-GCB	*P*-value	GCB	Non-GCB	*P*-value	GCB	Non-GCB	*P*-value	GCB	Non-GCB	*P*-value
Age (in years)												
≤ 60	0.04 ± 0.05	0.12 ± 0.08	0.54	0.09 ± 0.09	0.04 ± 0.05	0.12	0.05 ± 0.08	0.18 ± 0.14	0.87	0.04 ± 0.05	0.20 ± 0.11	0.29
> 60	0.04 ± 0.07	0.11 ± 0.09		0.11 ± 0.12	0.03 ± 0.04		0.06 ± 0.10	0.18 ± 0.18		0.05 ± 0.07	0.18 ± 0.13	
Gender												
Female	0.04 ± 0.06	0.11 ± 0.09	0.68	0.11 ± 0.12	0.04 ± 0.04	0.46	0.06 ± 0.09	0.17 ± 0.14	0.47	0.05 ± 0.07	0.19 ± 0.11	0.68
Male	0.04 ± 0.06	0.12 ± 0.09		0.09 ± 0.08	0.03 ± 0.04		0.06 ± 0.09	0.19 ± 0.18		0.04 ± 0.06	0.19 ± 0.13	
Ann Arbor stages												
I/II	0.05 ± 0.07	0.12 ± 0.10	0.62	0.09 ± 0.07	0.03 ± 0.04	0.19	0.07 ± 0.11	0.18 ± 0.17	0.60	0.05 ± 0.07	0.18 ± 0.11	0.25
III/IV	0.04 ± 0.05	0.12 ± 0.08		0.11 ± 0.12	0.03 ± 0.04		0.05 ± 0.08	0.18 ± 0.16		0.04 ± 0.06	0.20 ± 0.12	
B symptoms												
Absent	0.05 ± 0.04	0.13 ± 0.10	0.86	0.09 ± 0.09	0.04 ± 0.05	0.04*	0.07 ± 0.11	0.21 ± 0.20	0.43	0.04 ± 0.06	0.20 ± 0.12	0.78
Present	0.03 ± 0.08	0.11 ± 0.08		0.12 ± 0.12	0.03 ± 0.03		0.05 ± 0.67	0.16 ± 0.12		0.05 ± 0.07	0.18 ± 0.11	
LDH serum levels												
Normal	0.04 ± 0.06	0.11 ± 0.08	0.30	0.11 ± 0.11	0.03 ± 0.04	0.38	0.06 ± 0.09	0.17 ± 0.14	0.45	0.05 ± 0.07	0.19 ± 0.12	0.64
High	0.04 ± 0.06	0.13 ± 0.10		0.10 ± 0.10	0.04 ± 0.04		0.06 ± 0.09	0.19 ± 0.19		0.04 ± 0.06	0.19 ± 0.12	
Bulk												
≥ 2 cm	0.04 ± 0.06	0.12 ± 0.09	0.17	0.12 ± 0.12	0.04 ± 0.04	0.16	0.05 ± 0.09	0.20 ± 0.19	0.20	0.04 ± 0.07	0.19 ± 0.12	0.78
< 2 cm	0.04 ± 0.06	0.10 ± 0.08		0.08 ± 0.07	0.03 ± 0.03		0.06 ± 0.09	0.16 ± 0.11		0.04 ± 0.06	0.18 ± 0.12	
ECOG score												
0–1	0.05 ± 0.07	0.12 ± 0.09	0.30	0.10 ± 0.10	0.03 ± 0.04	0.86	0.07 ± 0.10	0.19 ± 0.18	0.79	0.05 ± 0.07	0.19 ± 0.11	0.32
≥ 2	0.03 ± 0.03	0.12 ± 0.10		0.10 ± 0.11	0.03 ± 0.05		0.03 ± 0.03	0.17 ± 0.15		0.02 ± 0.02	0.19 ± 0.13	

*: *P* ≤ 0.05

### ROC curve analysis

**Fig. 5:**
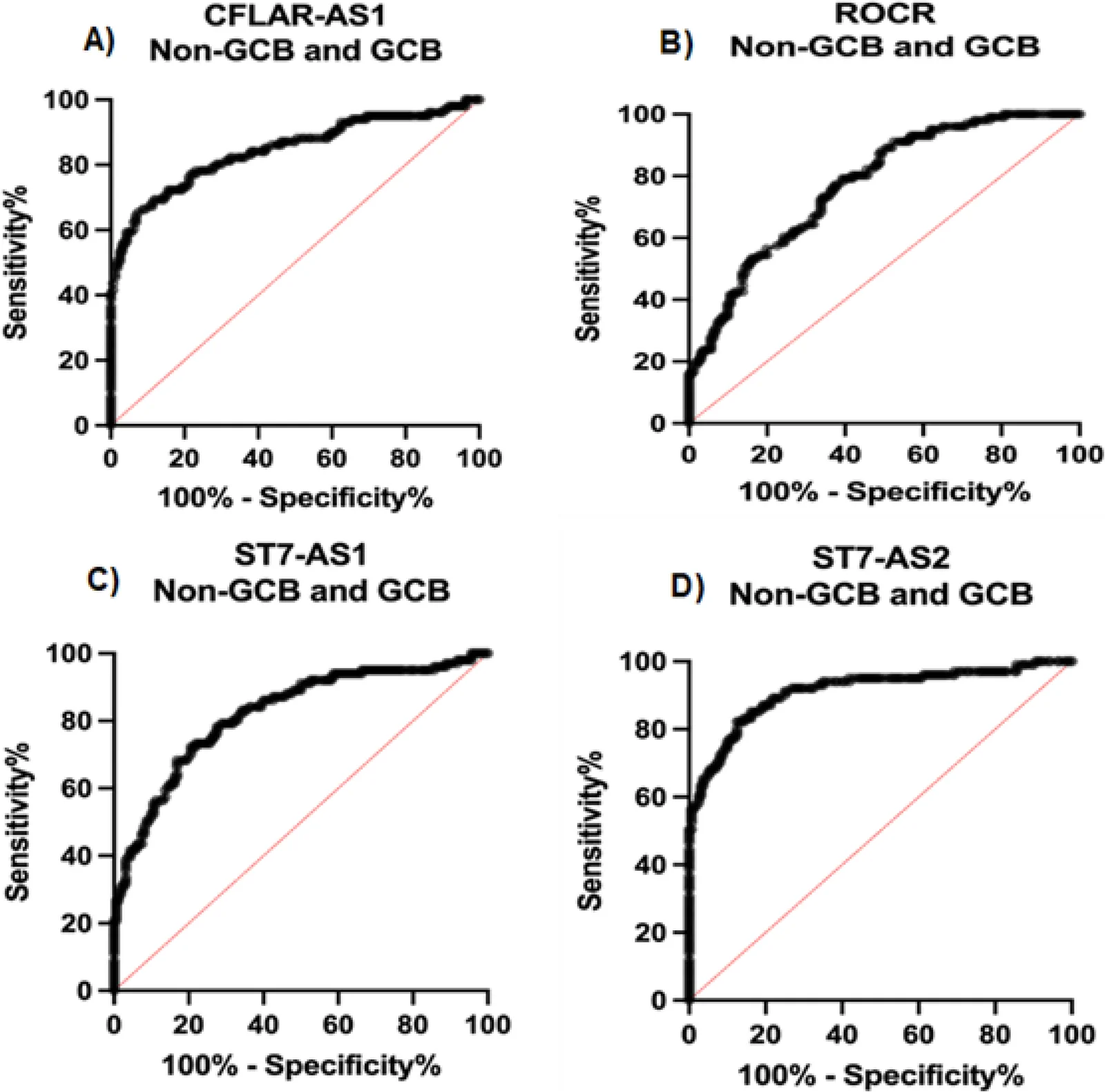
ROC curve analysis of the four lncRNAs for discriminating between GCB and non-GCB DLBCL subtypes. **A**) CFLAR-AS1 (AUC = 0.77, 95% CI: 0.7162–0.8283); **B**) ROCR (AUC = 0.84, 95% CI: 0.7913–0.8976); **C**) ST7-AS1 (AUC = 0.82, 95% CI: 0.7676–0.8747); **D**) ST7-AS2 (AUC = 0.91, 95% CI: 0.8729–0.9508)

The ROC curve was used to assess the discriminative power across the independent clinical validation cohort to evaluate these four lncRNAs. With *p* < 0.0001, the algorithm’s AUC was 91% for ST7-AS2 [95% CI: 0.8729–0.9508; sensitivity = 82%, specificity = 87%], 84% for CFLAR-AS1 [95% CI: 0.7913–0.8976; sensitivity = 77%, specificity = 78%], 82% for ST7-AS1 [95% CI: 0.7676–0.8747; sensitivity = 73%, specificity = 78%], and 77% for ROCR [95% CI: 0.7162–0.8283; sensitivity = 72%, specificity = 66%] (Fig. 5). These findings indicate strong discriminatory power for ST7-AS2, CFLAR-AS1, ST7-AS1, and ROCR, respectively, and suggest their potential as diagnostic biomarkers for classifying DLBCL subtypes.

## Discussion

DLBCL, a prevalent and aggressive lymphoma with extensive heterogeneity, can be primarily categorized into GCB and non-GCB subtypes [32]. The genetic characteristics of DLBCL have been extensively investigated in recent years, expanding our understanding of its molecular etiology [33], including differential histomorphology, clinical features, and epigenetics [32]. LncRNAs can regulate gene transcription, post-transcriptional modifications, and epigenetics, playing a role in the genesis and progression of diseases [34]. Given that several studies have highlighted the importance of lncRNAs in DLBCL development, progression, and treatment resistance [35], aberrant lncRNA expressions linked to clinical features or poor prognosis could serve as potential therapeutic targets or biomarkers for DLBCL [36].

The aim of the current research was to identify lncRNAs with the potential to be used as biomarkers for DLBCL diagnosis and to distinguish between GCB and non-GCB subtypes. Four unique lncRNAs (ROCR, CFLAR-AS1, ST7-AS1, and ST7-AS2) were selected via screening a previously published dataset in GEO [19], based on their significantly different expression between GCB and non-GCB subtypes and to gain insight into the biological processes and signaling pathways potentially associated with the identified lncRNAs, GO and KEGG enrichment analyses were performed using their co-expressed genes. Although using a cell line dataset provides a controlled, homogeneous environment without the background noise of other healthy cells, cell lines lack a natural tissue microenvironment; therefore, validating these candidates directly in clinical samples was necessary to prove their real-world relevance. Then, the expression levels of these lncRNAs were examined in DLBCL patients and controls, and further analysis was conducted in the subtypes. The results demonstrate significant associations between the expressions of all lncRNAs and DLBCL. Moreover, there were significantly elevated expression levels of all lncRNAs in non-GCB patients compared to GCB patients. Based on the ROC curve, our examined lncRNAs serve as potential biomarkers for the diagnosis of DLBCL and for distinguishing between the two subtypes, particularly ST7-AS2, which has a 91% chance of correctly classifying samples into GCB and non-GCB subtypes based on its expression.

Previous research demonstrated that ROCR regulates the expression of the transcription factor SOX9 in basal-like breast cancer cells and mesenchymal stem cells (MSCs), stimulating proliferation. Further studies have shown that ROCR recruits RNA polymerase II and increases H3K4me3 levels to trigger SOX9 transcription, elucidating the mechanism behind SOX9 transcriptional activity [30, 37]. The functional investigation revealed that RNA interference reduces ROCR, which disrupts MSC chondrogenesis, suggesting that ROCR may be involved in chondrogenic differentiation [30]. Bjørklund et al. found that ROCR has strong potential for distinguishing between patients with Luminal A and B breast cancer and is correlated with ER+ prognosis [38]. A network study of skin genes associated with Systemic Sclerosis by Roberson et al. revealed that SOX9-AS1 and ROCR, adjacent to the SOX9 locus, were identified as strongly connected, “hub-like” genes in the network, and they were correlated with clinical characteristics [39]. Although enrichment analysis did not identify a specific immune-related signature comparable to the other lncRNAs, the consistent upregulation of ROCR in our cohort suggests that it may contribute to DLBCL biology through mechanisms distinct from immune signaling.

Hu et al. reported that circulating CFLAR-AS1 might be a potential biomarker for predicting the early stages of esophageal squamous cell carcinoma. They also demonstrated that circulating CFLAR-AS1 was associated with a poor prognosis for patients with esophageal cancer after surgery [26]. According to studies, CFLAR-AS1 is involved in immune-related pathways such as the NF-κB and TNF signaling pathways [23]. Studies have shown that CFLAR-AS1 is critical for transcription, cell cycle development, and survival [40]. Another investigation revealed that increased CFLAR expression in the ABC subtype may promote cell proliferation. A different analysis found that CFLAR enrichment influences the regulation of satellite cell proliferation [41]. In addition, our enrichment analysis suggests that CFLAR-AS1 is associated with adaptive immune responses, T-cell activation, Th17 differentiation, and cytokine-mediated signaling pathways, including JAK–STAT, TNF, and Toll-like receptor signaling. Given the central role of TNF and Toll-like receptor signaling in NF-κB activation, these findings suggest that CFLAR-AS1 may be associated with inflammatory signaling networks implicated in non-GCB DLBCL.

According to several studies, there is a substantial association between ST7-AS1 and laryngeal squamous cell carcinoma, lung adenocarcinoma, and human gliomas. Increased expression of ST7-AS1 is linked to advanced cancers, metastasis, and poor prognosis for cancer patients [27, 42]. Hu et al. showed considerable upregulation of ST7-AS1 in the tissues of patients with lung adenocarcinoma. This upregulation was associated with tumors having higher TNM stages or positive lymph node metastases and indicated shorter overall survival for these patients [42]. Bioinformatics research and high-throughput RNA sequencing showed that ST7-AS1 expression was significantly lower in breast cancer, and that decreased ST7-AS1 expression was associated with pathological features [27]. Cai et al. demonstrated that ST7-AS1 levels were elevated in patients with gastric cancer and correlated with tumor size and stage, suggesting a carcinogenic role for ST7-AS1 in gastric cancer. Furthermore, ST7-AS1 knockdown reduced the ability of gastric cancer cells to proliferate, migrate, and invade; it also arrested the cell cycle and induced apoptosis [43]. The cytoplasm of papillary thyroid cancer cells was identified as the principal location for ST7-AS1. Functional tests showed that overexpression of ST7-AS1 enhanced apoptosis of papillary thyroid cancer cells while inhibiting their viability and proliferation [44]. Qi et al. demonstrated that cervical cancer tissues and cell lines have increased ST7-AS1 expression. Moreover, in individuals with cervical cancer, ST7-AS1 overexpression was substantially associated with deep cervical invasion, frequent lymph node metastases, and shorter overall survival [45]. The enrichment of Th17-related immune processes and T-cell differentiation suggests that ST7-AS1 may be involved in immune regulation in DLBCL. Combined with its elevated expression in the non-GCB subtype, these findings support a potential association between ST7-AS1 and inflammatory signaling networks characteristic of non-GCB DLBCL.

ST7-AS2 may target miR-106a, which is differentially expressed in colorectal cancer [46]. However, survival analyses showed that increased lymphoma mortality was linked to plasma levels of miR-106a/b [47]. Du et al. demonstrated a substantial correlation between ST7-AS2 expression and survival rates in esophageal cancer patients. They also suggested a potential link between alcohol-related esophageal cancer and ST7-AS2 [48]. Another study found that ST7-AS2 expression was elevated in triple-negative breast cancer and that it correlated with the TNM stage of this disease [13]. Notably, all three selected lncRNAs demonstrated enrichment for adaptive immune responses and Th17-related pathways, while ST7-AS2 additionally showed enrichment of NF-κB, TNF, JAK–STAT, and PD-L1/PD-1 signaling. This broader immune-related enrichment suggests that ST7-AS2 may have a more central association with inflammatory and immune regulatory networks in DLBCL than CFLAR-AS1 or ST7-AS1.

The Hans algorithm remains the most widely used method for DLBCL subtype classification; however, it shows approximately 15–20% discordance with gene expression profiling, the reference standard [49]. In contrast, qRT-PCR-based lncRNA expression analysis provides an objective and quantitative molecular assessment. Although our lncRNA panel, particularly ST7-AS2, showed good discriminatory performance, future studies are needed to determine whether combining lncRNA profiling with the Hans algorithm can improve DLBCL subtype classification.

However, the limited sample size and reliance on a single GEO cell line dataset rather than patient-derived transcriptomic data for lncRNA identification should be considered limitations of this study. While cell lines provide a controlled experimental framework, they lack the complex tumor microenvironment and stromal interactions present in primary DLBCL tissues, which may influence lncRNA expression patterns and limit the generalizability of our discovery-phase findings. A larger and more diverse cohort may provide greater accuracy and stronger statistical power. The exclusively Iranian composition of our cohort may limit generalizability to other populations, and the cross-sectional design precludes evaluation of prognostic value or longitudinal expression changes. GO and KEGG enrichment analyses suggested that CFLAR-AS1, ST7-AS1, and ST7-AS2 are associated with adaptive immune responses, Th17 cell differentiation, and immune-related signaling pathways, including JAK–STAT, TNF, NF-κB, and PD-L1/PD-1 signaling. However, these functional annotations are correlative and derived from co-expressed protein-coding genes rather than direct experimental evidence, and thus cannot establish causal relationships or reveal specific molecular mechanisms. Nevertheless, further in vitro and in vivo functional studies are required to validate these associations and elucidate the roles of these lncRNAs in DLBCL. Loss and gain of function experiments, RNA immunoprecipitation, and xenograft models will be necessary to confirm their functions and identify downstream targets. Furthermore, multi-omics approaches, including transcriptomics, proteomics, and epigenomics, may provide a more comprehensive molecular characterization of DLBCL.

## Conclusion

In conclusion, this study screened the GSE207388 dataset in DLBCL and identified four lncRNAs CFLAR-AS1, ROCR, ST7-AS1, and ST7-AS2 for further investigation. All four lncRNAs were upregulated in DLBCL patients and were associated with the non-GCB subtype compared with the GCB subtype. ROC curve analysis demonstrated their strong diagnostic performance, particularly ST7-AS2, with a 91% chance of correctly classifying samples into GCB and non-GCB subtypes. GO and KEGG enrichment analyses suggested that CFLAR-AS1, ST7-AS1, and ST7-AS2 are associated with adaptive immune responses, Th17 cell differentiation, and immune-related signaling pathways, including JAK–STAT, TNF, NF-κB, and PD-L1/PD-1 signaling. These findings provide a foundation for further investigation into the role of these lncRNAs in DLBCL biology and their potential as diagnostic biomarkers. Validation in larger, multi-center cohorts with longitudinal follow-up and functional studies will be essential to translate these findings into clinical applications.

## Supplementary Information

Supplementary Material 1.

## Data Availability

The datasets used and/or analyzed during the current study are available from the corresponding and first authors on reasonable request.

## Abbreviations

DLBCL: Diffuse Large B-cell lymphoma
GCB: Germinal Center B-cell
non-GCB/ABC: non-Germinal Center/Activated B-cell
DEGs: Differentially Expressed Genes
lncRNA: Long non-coding RNA
HL: Hodgkin's Lymphoma
NHL: Non-Hodgkin's Lymphoma
BCR: B-cell receptor
GEO: Gene Expression Omnibus
ESCC: Esophageal Squamous Cell Carcinoma
ROCR: Regulator of Chondrogenesis RNA
CFLAR-AS1: Caspase 8 and Fas-associated via death domain like apoptosis regulator antisense RNA 1
ST7-AS1: ST7 antisense RNA1
ST7-AS2: ST7 antisense RNA2
qRT-PCR: Quantitative Real-Time Reverse Transcription Polymerase Chain Reaction
cDNA: Complementary DNA
AUC: Areas Under Curves
ROC: Receiver Operating Characteristic
