# Supplementary material for: Expression profiles of long non-coding RNAs ROCR, CFLAR-AS1, ST7-AS1, and ST7-AS2 in diffuse large B-cell lymphoma: a case-control study focusing on GCB and non-GCB subtypes
Source: BMC Med Genomics. 2026 Aug 10;19:170. doi: 10.1186/s12920-026-02452-0 (PMC13737518; doi:10.1186/s12920-026-02452-0)
Supplement: Supplementary file 1 [file 12920_2026_2452_MOESM1_ESM.docx]

|  | **GeneID** | **Symbol** | **Description** | **log2(fold change)** | **-log10(Pvalue)** |
| --- | --- | --- | --- | --- | --- |
| 1. | **8863** | PER3 | period circadian regulator 3 | 3.21 | 3.085 |
| 2. | **80176** | SPSB1 | splA/ryanodine receptor domain and SOCS box containing 1 | 4.346 | 3.82 |
| 3. | **3339** | HSPG2 | heparan sulfate proteoglycan 2 | -3.991 | 3.342 |
| 4. | **2810** | SFN | stratifin | -3.25 | 4.994 |
| 5. | **2268** | FGR | FGR proto-oncogene, Src family tyrosine kinase | 4.018 | 4.302 |
| 6. | **79830** | ZMYM1 | zinc finger MYM-type containing 1 | 2.117 | 9.982 |
| 7. | **149478** | BTBD19 | BTB domain containing 19 | -3.054 | 7.821 |
| 8. | **3725** | JUN | Jun proto-oncogene, AP-1 transcription factor subunit | -2.379 | 3.222 |
| 9. | **107985037** | LOC107985037 |  | -5.065 | 3.086 |
| 10. | **79739** | TTLL7 | tubulin tyrosine ligase like 7 | -4.416 | 3.77 |
| 11. | **2634** | GBP2 | guanylate binding protein 2 | -4.44 | 6.5 |
| 12. | **112268267** | LOC112268267 | uncharacterized LOC112268267 | -3.604 | 5.281 |
| 13. | **7049** | TGFBR3 | transforming growth factor beta receptor 3 | -4.935 | 4.903 |
| 14. | **57535** | ELAPOR1 | endosome-lysosome associated apoptosis and autophagy regulator 1 | 3.841 | 4.262 |
| 15. | **6277** | S100A6 | S100 calcium binding protein A6 | -5.028 | 4.163 |
| 16. | **9047** | SH2D2A | SH2 domain containing 2A | -2.903 | 3.517 |
| 17. | **57823** | SLAMF7 | SLAM family member 7 | -5.166 | 5.242 |
| 18. | **4688** | NCF2 | neutrophil cytosolic factor 2 | -4.746 | 8.792 |
| 19. | **23127** | COLGALT2 | collagen beta(1-O)galactosyltransferase 2 | -3.521 | 3.2 |
| 20. | **8934** | RAB29 | RAB29, member RAS oncogene family | -3.047 | 7.182 |
| 21. | **105372857** | LOC105372857 | uncharacterized LOC105372857 | -3.658 | 3.126 |
| 22. | **5284** | PIGR | polymeric immunoglobulin receptor | -4.599 | 4.612 |
| 23. | **467** | ATF3 | activating transcription factor 3 | -3.565 | 5.017 |
| 24. | **400941** | LINC00487 | long intergenic non-protein coding RNA 487 | 9.229 | 10.136 |
| 25. | **9781** | RNF144A | ring finger protein 144A | 5.529 | 4.248 |
| 26. | **105373430** | LOC105373430 | uncharacterized LOC105373430 | 6.892 | 9.982 |
| 27. | **100506457** | MIR3681HG | MIR3681 host gene | 6.318 | 7.215 |
| 28. | **105373433** | LOC105373433 | uncharacterized LOC105373433 | 8.235 | 9.086 |
| 29. | **28951** | TRIB2 | tribbles pseudokinase 2 | 3.49 | 3.761 |
| 30. | **7447** | VSNL1 | visinin like 1 | -3.944 | 7.06 |
| 31. | **56660** | KCNK12 | potassium two pore domain channel subfamily K member 12 | 3.421 | 5.28 |
| 32. | **114800** | CCDC85A | coiled-coil domain containing 85A | 8.572 | 10.424 |
| 33. | **105374790** | LOC105374790 | uncharacterized LOC105374790 | -2.177 | 3.205 |
| 34. | **28914** | IGKV3-11 | immunoglobulin kappa variable 3-11 | -4.241 | 3.532 |
| 35. | **28912** | IGKV3-20 | immunoglobulin kappa variable 3-20 | -8.788 | 14.503 |
| 36. | **28921** | IGKV2-28 | immunoglobulin kappa variable 2-28 | 5.635 | 3.67 |
| 37. | **100216479** | FAR2P2 | fatty acyl-CoA reductase 2 pseudogene 2 | 6.024 | 8.238 |
| 38. | **3800** | KIF5C | kinesin family member 5C | 2.699 | 3.258 |
| 39. | **130576** | LYPD6B | LY6/PLAUR domain containing 6B | 6.176 | 3.731 |
| 40. | **11227** | GALNT5 | polypeptide N-acetylgalactosaminyltransferase 5 | -3.136 | 3.906 |
| 41. | **6326** | SCN2A | sodium voltage-gated channel alpha subunit 2 | -6.033 | 8.243 |
| 42. | **107985968** | LOC107985968 | uncharacterized LOC107985968 | -6.277 | 4.971 |
| 43. | **64859** | NABP1 | nucleic acid binding protein 1 | -2.52 | 6.062 |
| 44. | **105747689** | NABP1-OT1 | replaced by ID 64859 | -3.233 | 6.245 |
| 45. | **101927454** | LOC101927454 | uncharacterized LOC101927454 | 2.213 | 6.652 |
| 46. | **57520** | HECW2 | HECT, C2 and WW domain containing E3 ubiquitin protein ligase 2 | 2.582 | 3.125 |
| 47. | **8837** | CFLAR | CASP8 and FADD like apoptosis regulator | -2.541 | 5.651 |
| 48. | **65072** | CFLAR-AS1 | CFLAR antisense RNA 1 | -2.54 | 4.996 |
| 49. | **8828** | NRP2 | neuropilin 2 | 4.159 | 3.966 |
| 50. | **103689911** | CATIP-AS2 | CATIP antisense RNA 2 | 4.803 | 4.458 |
| 51. | **5133** | PDCD1 | programmed cell death 1 | -4.871 | 4.457 |
| 52. | **8536** | CAMK1 | calcium/calmodulin dependent protein kinase I | 3.635 | 4.488 |
| 53. | **105376956** | LOC105376956 | uncharacterized LOC105376956 | -3.522 | 3.352 |
| 54. | **100505696** | SH3BP5-AS1 | SH3BP5 antisense RNA 1 | -2.221 | 4.03 |
| 55. | **9467** | SH3BP5 | SH3 domain binding protein 5 | -2.452 | 3.202 |
| 56. | **389119** | INKA1 | inka box actin regulator 1 | 3.471 | 6.775 |
| 57. | **776** | CACNA1D | calcium voltage-gated channel subunit alpha1 D | -4.988 | 6.988 |
| 58. | **54899** | PXK | PX domain containing serine/threonine kinase like | 2.029 | 7.455 |
| 59. | **105377106** | LOC105377106 | uncharacterized LOC105377106 | 2.491 | 6.176 |
| 60. | **8618** | CADPS | calcium dependent secretion activator | 4.93 | 3.941 |
| 61. | **285286** | LINC00877 | long intergenic non-protein coding RNA 877 | 3.892 | 3.301 |
| 62. | **131909** | FAM172BP | family with sequence similarity 172 member B, pseudogene | -5.158 | 4.313 |
| 63. | **64332** | NFKBIZ | NFKB inhibitor zeta | -2.928 | 3.977 |
| 64. | **152404** | IGSF11 | immunoglobulin superfamily member 11 | -4.37 | 4.226 |
| 65. | **56941** | HMCES | 5-hydroxymethylcytosine binding, ES cell specific | 3.311 | 3.823 |
| 66. | **105374140** | LOC105374140 | uncharacterized LOC105374140 | -2.133 | 3.066 |
| 67. | **4311** | MME | membrane metalloendopeptidase | 5.055 | 8.669 |
| 68. | **93556** | EGFEM1P | EGF like and EMI domain containing 1, pseudogene | -6.629 | 4.708 |
| 69. | **339926** | EHHADH-AS1 | EHHADH antisense RNA 1 | -4.607 | 4.39 |
| 70. | **1962** | EHHADH | enoyl-CoA hydratase and 3-hydroxyacyl CoA dehydrogenase | -4.636 | 4.759 |
| 71. | **105374266** | LINC01991 | long intergenic non-protein coding RNA 1991 | 4.206 | 3.23 |
| 72. | **4026** | LPP | LIM domain containing preferred translocation partner in lipoma | 2.65 | 3.051 |
| 73. | **257313** | UTS2B | urotensin 2B | -3.96 | 3.842 |
| 74. | **3627** | CXCL10 | C-X-C motif chemokine ligand 10 | -7.4 | 15.093 |
| 75. | **5602** | MAPK10 | mitogen-activated protein kinase 10 | 4.651 | 5.734 |
| 76. | **9429** | ABCG2 | ATP binding cassette subfamily G member 2 (Junior blood group) | -5.391 | 4.405 |
| 77. | **166929** | SGMS2 | sphingomyelin synthase 2 | -3.055 | 3.187 |
| 78. | **2247** | FGF2 | fibroblast growth factor 2 | -2.285 | 3.223 |
| 79. | **55534** | MAML3 | mastermind like transcriptional coactivator 3 | 3.312 | 3.532 |
| 80. | **8821** | INPP4B | inositol polyphosphate-4-phosphatase type II B | -8.134 | 13.557 |
| 81. | **4086** | SMAD1 | SMAD family member 1 | 6.11 | 4.513 |
| 82. | **166614** | DCLK2 | doublecortin like kinase 2 | -5.125 | 4.507 |
| 83. | **56884** | FSTL5 | follistatin like 5 | -5.443 | 4.164 |
| 84. | **10723** | SLC12A7 | solute carrier family 12 member 7 | -7.174 | 9.623 |
| 85. | **3672** | ITGA1 | integrin subunit alpha 1 | -5.865 | 9.291 |
| 86. | **3673** | ITGA2 | integrin subunit alpha 2 | -2.878 | 3.536 |
| 87. | **107986348** | LOC107986348 |  | 4.563 | 4.672 |
| 88. | **107986347** | LOC107986347 | uncharacterized LOC107986347 | 5.081 | 3.872 |
| 89. | **105378988** | LOC105378988 | uncharacterized LOC105378988 | 4.537 | 3.209 |
| 90. | **107986350** | LOC107986350 | uncharacterized LOC107986350 | 4.411 | 4.161 |
| 91. | **25859** | PART1 | prostate androgen-regulated transcript 1 | 3.553 | 4.582 |
| 92. | **22936** | ELL2 | elongation factor for RNA polymerase II 2 | -3.827 | 4.966 |
| 93. | **353376** | TICAM2 | TIR domain containing adaptor molecule 2 | -2.326 | 5.033 |
| 94. | **5098** | PCDHGC3 | protocadherin gamma subfamily C, 3 | 3.209 | 5.013 |
| 95. | **56097** | PCDHGC5 | protocadherin gamma subfamily C, 5 | 2.361 | 3.355 |
| 96. | **3702** | ITK | IL2 inducible T cell kinase | 4.808 | 5.146 |
| 97. | **11063** | SOX30 | SRY-box transcription factor 30 | -3.283 | 3.192 |
| 98. | **100128340** | LOC100128340 | uncharacterized LOC100128340 | -3.324 | 4.687 |
| 99. | **153579** | BTNL9 | butyrophilin like 9 | 4.948 | 4.946 |
| 100. | **105374875** | LOC105374875 |  | -4.439 | 8.109 |
| 101. | **2296** | FOXC1 | forkhead box C1 | -3.957 | 3.135 |
| 102. | **84830** | ADTRP | androgen dependent TFPI regulating protein | -7.951 | 12.763 |
| 103. | **54438** | GFOD1 | glucose-fructose oxidoreductase domain containing 1 | 2.487 | 3.065 |
| 104. | **105374995** | LOC105374995 |  | -2.567 | 7.227 |
| 105. | **105379695** | IER3-AS1 | IER3 antisense RNA 1 | -2.842 | 5.28 |
| 106. | **8870** | IER3 | immediate early response 3 | -3.222 | 5.509 |
| 107. | **3127** | HLA-DRB5 | major histocompatibility complex, class II, DR beta 5 | 4.095 | 3.98 |
| 108. | **101929770** | LOC101929770 | uncharacterized LOC101929770 | -2.167 | 3.228 |
| 109. | **105377891** | LOC105377891 | uncharacterized LOC105377891 | 5.117 | 3.852 |
| 110. | **639** | PRDM1 | PR/SET domain 1 | -4.234 | 4.564 |
| 111. | **105377927** | LOC105377927 | uncharacterized LOC105377927 | 2.978 | 4.398 |
| 112. | **101927686** | LINC02541 | long intergenic non-protein coding RNA 2541 | -4.914 | 4.531 |
| 113. | **2697** | GJA1 | gap junction protein alpha 1 | -6.292 | 6.231 |
| 114. | **5796** | PTPRK | protein tyrosine phosphatase receptor type K | -4.721 | 3.536 |
| 115. | **5169** | ENPP3 | ectonucleotide pyrophosphatase/phosphodiesterase 3 | 4.081 | 3.249 |
| 116. | **8876** | VNN 1.00 | vanin 1 | -6.499 | 5.171 |
| 117. | **84624** | FNDC1 | fibronectin type III domain containing 1 | 6.087 | 3.086 |
| 118. | **221935** | SDK1 | sidekick cell adhesion molecule 1 | 4.665 | 3.088 |
| 119. | **105375153** | LOC105375153 |  | 4.04 | 3.774 |
| 120. | **105375172** | LOC105375172 | uncharacterized LOC105375172 | -4.921 | 3.483 |
| 121. | **80820** | EEPD1 | endonuclease/exonuclease/phosphatase family domain containing 1 | 3.096 | 3.184 |
| 122. | **313** | AOAH | acyloxyacyl hydrolase | -4.828 | 8.387 |
| 123. | **107** | ADCY1 | adenylate cyclase 1 | -3.115 | 3.761 |
| 124. | **101929086** | LINC01447 | long intergenic non-protein coding RNA 1447 | 4.809 | 5.182 |
| 125. | **107986798** | LOC107986798 |  | -2.269 | 4.004 |
| 126. | **101929322** | LOC101929322 | integrator complex subunit 4 pseudogene | 6.662 | 9.137 |
| 127. | **948** | CD36 | CD36 molecule | -9.149 | 18.811 |
| 128. | **6856** | SYPL1 | synaptophysin like 1 | 2.141 | 4.962 |
| 129. | **93653** | ST7-AS1 | ST7 antisense RNA 1 | -2.071 | 3.824 |
| 130. | **7982** | ST7 | suppression of tumorigenicity 7 | -2.179 | 3.88 |
| 131. | **93654** | ST7-AS2 | ST7 antisense RNA 2 | -2.273 | 4.635 |
| 132. | **93655** | ST7-OT3 | ST7 overlapping transcript 3 | -2.048 | 3.517 |
| 133. | **102724660** | HILPDA-AS1 | HILPDA antisense RNA 1 | -2.952 | 7.178 |
| 134. | **29923** | HILPDA | hypoxia inducible lipid droplet associated | -2.773 | 5.013 |
| 135. | **101928466** | LOC101928466 | uncharacterized LOC101928466 | -4.035 | 5.395 |
| 136. | **285966** | TCAF2 | TRPM8 channel associated factor 2 | -3.307 | 3.05 |
| 137. | **653691** | TCAF2P1 | TRPM8 channel associated factor 2 pseudogene 1 | -3.376 | 4.023 |
| 138. | **154761** | LOC154761 | family with sequence similarity 115, member C pseudogene | -3.276 | 3.29 |
| 139. | **112267988** | LOC112267988 | uncharacterized LOC112267988 | -4.126 | 5.087 |
| 140. | **23362** | PSD3 | pleckstrin and Sec7 domain containing 3 | 6.169 | 5.137 |
| 141. | **11178** | LZTS1 | leucine zipper tumor suppressor 1 | 5.415 | 4.08 |
| 142. | **3574** | IL7 | interleukin 7 | -5.662 | 17.888 |
| 143. | **101241902** | C4orf46P3 | C4orf46 pseudogene 3 | -3.318 | 4.946 |
| 144. | **105375914** | LOC105375914 | uncharacterized LOC105375914 | -7.884 | 18.494 |
| 145. | **105375913** | LOC105375913 | uncharacterized LOC105375913 | -8.397 | 22.721 |
| 146. | **55824** | PAG1 | phosphoprotein membrane anchor with glycosphingolipid microdomains 1 | 3.35 | 5.281 |
| 147. | **105375924** | LOC105375924 | uncharacterized LOC105375924 | 4.761 | 8.987 |
| 148. | **105375650** | MIR3150BHG | MIR3150B host gene | 3.356 | 5.064 |
| 149. | **79666** | PLEKHF2 | pleckstrin homology and FYVE domain containing 2 | 2.041 | 4.542 |
| 150. | **5168** | ENPP2 | ectonucleotide pyrophosphatase/phosphodiesterase 2 | -6.638 | 9.754 |
| 151. | **29126** | CD274 | CD274 molecule | -4.158 | 8.308 |
| 152. | **100132154** | LOC100132154 | ankyrin repeat domain 30B pseudogene | -4.209 | 3.223 |
| 153. | **2650** | GCNT1 | glucosaminyl (N-acetyl) transferase 1 | 3.467 | 7.257 |
| 154. | **55363** | HEMGN | hemogen | 4.161 | 3.07 |
| 155. | **8328** | GFI1B | growth factor independent 1B transcriptional repressor | 5.005 | 4.134 |
| 156. | **105376314** | LOC105376314 | uncharacterized LOC105376314 | -4.346 | 3.13 |
| 157. | **105376323** | LINC02846 | long intergenic non-protein coding RNA 2846 | -3.549 | 5.146 |
| 158. | **105376413** | LOC105376413 | uncharacterized LOC105376413 | 3.234 | 3.505 |
| 159. | **105376412** | LOC105376412 | uncharacterized LOC105376412 | 3.862 | 3.848 |
| 160. | **55691** | FRMD4A | FERM domain containing 4A | -2.265 | 4.177 |
| 161. | **9200** | HACD1 | 3-hydroxyacyl-CoA dehydratase 1 | -5.003 | 3.093 |
| 162. | **8187** | ZNF239 | zinc finger protein 239 | 8.047 | 6.232 |
| 163. | **288** | ANK3 | ankyrin 3 | 5.765 | 9.436 |
| 164. | **10974** | ADIRF | adipogenesis regulatory factor | -4.517 | 3.96 |
| 165. | **84795** | PYROXD2 | pyridine nucleotide-disulphide oxidoreductase domain 2 | -3.547 | 7.625 |
| 166. | **105378515** | LOC105378515 | uncharacterized LOC105378515 | 9.561 | 8.953 |
| 167. | **105378531** | LINC02641 | long intergenic non-protein coding RNA 2641 | -3.348 | 3.532 |
| 168. | **64077** | LHPP | phospholysine phosphohistidine inorganic pyrophosphate phosphatase | 2.198 | 5.741 |
| 169. | **101** | ADAM8 | ADAM metallopeptidase domain 8 | -3.107 | 4.898 |
| 170. | **2620** | GAS2 | growth arrest specific 2 | -3.528 | 7.665 |
| 171. | **25841** | ABTB2 | ankyrin repeat and BTB domain containing 2 | -2.53 | 6.724 |
| 172. | **105376626** | LOC105376626 | uncharacterized LOC105376626 | -3.869 | 3.649 |
| 173. | **6688** | SPI1 | Spi-1 proto-oncogene | 2.25 | 5.81 |
| 174. | **107984341** | LOC107984341 | uncharacterized LOC107984341 | -2.016 | 4.149 |
| 175. | **374407** | DNAJB13 | DnaJ heat shock protein family (Hsp40) member B13 | -2.863 | 6.139 |
| 176. | **81544** | GDPD5 | glycerophosphodiester phosphodiesterase domain containing 5 | 3.53 | 4.193 |
| 177. | **105369389** | LOC105369389 | uncharacterized LOC105369389 | 3.431 | 4.392 |
| 178. | **4647** | MYO7A | myosin VIIA | -5.258 | 4.591 |
| 179. | **894** | CCND2 | cyclin D2 | -6.115 | 5.435 |
| 180. | **105369614** | LOC105369614 |  | 5.052 | 7.169 |
| 181. | **3742** | KCNA6 | potassium voltage-gated channel subfamily A member 6 | 3.526 | 4.361 |
| 182. | **100528032** | KLRC4-KLRK1 | KLRC4-KLRK1 readthrough | 6.029 | 7.822 |
| 183. | **22914** | KLRK1 | killer cell lectin like receptor K1 | 6.071 | 8.109 |
| 184. | **8302** | KLRC4 | killer cell lectin like receptor C4 | 5.749 | 4.711 |
| 185. | **3823** | KLRC3 | killer cell lectin like receptor C3 | 4.869 | 4.078 |
| 186. | **79365** | BHLHE41 | basic helix-loop-helix family member e41 | -5.452 | 5.37 |
| 187. | **107984461** | MRPS35-DT | MRPS35 divergent transcript | -2.734 | 3.616 |
| 188. | **283461** | C12orf40 | chromosome 12 open reading frame 40 | -2.952 | 3.04 |
| 189. | **112268095** | LOC112268095 | uncharacterized LOC112268095 | -3.023 | 4.214 |
| 190. | **4753** | NELL2 | neural EGFL like 2 | 6.134 | 5.182 |
| 191. | **11213** | IRAK3 | interleukin 1 receptor associated kinase 3 | -5.157 | 10.042 |
| 192. | **27345** | KCNMB4 | potassium calcium-activated channel subfamily M regulatory beta subunit 4 | 2.915 | 4.183 |
| 193. | **1848** | DUSP6 | dual specificity phosphatase 6 | 3.196 | 3.228 |
| 194. | **144535** | CFAP54 | cilia and flagella associated protein 54 | -4.257 | 4.42 |
| 195. | **3479** | IGF1 | insulin like growth factor 1 | -3.45 | 5.436 |
| 196. | **107984130** | LOC107984130 |  | -4.484 | 9.739 |
| 197. | **115811** | IQCD | IQ motif containing D | 2.091 | 3.517 |
| 198. | **100188949** | LINC00426 | long intergenic non-protein coding RNA 426 | 3.008 | 5.579 |
| 199. | **107984577** | LOC107984577 | uncharacterized LOC107984577 | 4.119 | 5.23 |
| 200. | **81617** | CAB39L | calcium binding protein 39 like | 2.731 | 4.231 |
| 201. | **2262** | GPC5 | glypican 5 | -5.398 | 5.472 |
| 202. | **10673** | TNFSF13B | TNF superfamily member 13b | -2.403 | 3.033 |
| 203. | **105370438** | LOC105370438 | uncharacterized LOC105370438 | -4.462 | 5.035 |
| 204. | **105370452** | LOC105370452 | uncharacterized LOC105370452 | 9.217 | 6.839 |
| 205. | **10979** | FERMT2 | FERM domain containing kindlin 2 | -5.503 | 10.624 |
| 206. | **105370502** | LOC105370502 | uncharacterized LOC105370502 | 2.343 | 4.262 |
| 207. | **3958** | LGALS3 | galectin 3 | -3.336 | 8.109 |
| 208. | **161145** | TMEM229B | transmembrane protein 229B | 3.065 | 4.488 |
| 209. | **101928075** | TTC9-DT | TTC9 divergent transcript | 4.572 | 5.65 |
| 210. | **23508** | TTC9 | tetratricopeptide repeat domain 9 | 3.831 | 6.037 |
| 211. | **105370572** | LOC105370572 | uncharacterized LOC105370572 | -5.63 | 4.604 |
| 212. | **55640** | FLVCR2 | FLVCR heme transporter 2 | -2.938 | 6.078 |
| 213. | **107984673** | LOC107984673 | uncharacterized LOC107984673 | -2.715 | 5.402 |
| 214. | **1152** | CKB | creatine kinase B | -4.196 | 7.516 |
| 215. | **3502** | IGHG3 | immunoglobulin heavy constant gamma 3 (G3m marker) | -3.903 | 3.334 |
| 216. | **6095** | RORA | RAR related orphan receptor A | -4.07 | 8.953 |
| 217. | **1138** | CHRNA5 | cholinergic receptor nicotinic alpha 5 subunit | -2.026 | 3.836 |
| 218. | **1136** | CHRNA3 | cholinergic receptor nicotinic alpha 3 subunit | -2.123 | 3.679 |
| 219. | **64714** | PDIA2 | protein disulfide isomerase family A member 2 | -3.847 | 3.415 |
| 220. | **26063** | DECR2 | 2,4-dienoyl-CoA reductase 2 | -2.236 | 3.679 |
| 221. | **197358** | NLRC3 | NLR family CARD domain containing 3 | 2.834 | 3.789 |
| 222. | **101929732** | LOC101929732 | uncharacterized LOC101929732 | 2.309 | 4.415 |
| 223. | **162073** | ITPRIPL2 | ITPRIP like 2 | 4.743 | 3.173 |
| 224. | **554206** | LOC554206 | leucine carboxyl methyltransferase 1 pseudogene | -2.731 | 4.345 |
| 225. | **388242** | LOC388242 | SAGA complex associated factor 29 pseudogene | 3.259 | 3.53 |
| 226. | **283897** | C16orf54 | chromosome 16 open reading frame 54 | 3.605 | 3.643 |
| 227. | **613038** | LOC613038 | SAGA complex associated factor 29 pseudogene | 3.307 | 3.634 |
| 228. | **107984867** | LOC107984867 | uncharacterized LOC107984867 | 4.447 | 3.033 |
| 229. | **404550** | C16orf74 | chromosome 16 open reading frame 74 | 5.056 | 3.667 |
| 230. | **57338** | JPH3 | junctophilin 3 | -2.245 | 3.204 |
| 231. | **388325** | SCIMP | SLP adaptor and CSK interacting membrane protein | 3.335 | 3.719 |
| 232. | **9423** | NTN1 | netrin 1 | -2.645 | 3.408 |
| 233. | **2886** | GRB7 | growth factor receptor bound protein 7 | -3.92 | 4.888 |
| 234. | **201181** | ZNF385C | zinc finger protein 385C | -3.672 | 15.82 |
| 235. | **6774** | STAT3 | signal transducer and activator of transcription 3 | -2.208 | 4.555 |
| 236. | **100885848** | PTGES3L | prostaglandin E synthase 3 like | -2.029 | 3.427 |
| 237. | **3131** | HLF | HLF transcription factor, PAR bZIP family member | -5.413 | 3.577 |
| 238. | **56155** | TEX14 | testis expressed 14, intercellular bridge forming factor | -2.703 | 3.918 |
| 239. | **105371849** | HEATR6-DT | HEATR6 divergent transcript | -5.573 | 13.145 |
| 240. | **645638** | WFDC21P | WAP four-disulfide core domain 21, pseudogene | -6.174 | 10.688 |
| 241. | **284021** | MILR1 | mast cell immunoglobulin like receptor 1 | 2.64 | 5.145 |
| 242. | **27091** | CACNG5 | calcium voltage-gated channel auxiliary subunit gamma 5 | 3.963 | 3.099 |
| 243. | **102723505** | ROCR | regulator of chondrogenesis RNA | 8.237 | 5.402 |
| 244. | **102606463** | LINC01152 | long intergenic non-protein coding RNA 1152 | 6.052 | 4.168 |
| 245. | **114804** | RNF157 | ring finger protein 157 | -3.544 | 3.271 |
| 246. | **9021** | SOCS3 | suppressor of cytokine signaling 3 | -5.501 | 4.391 |
| 247. | **284131** | ENDOV | endonuclease V | -2.718 | 4.39 |
| 248. | **644669** | LOC644669 | ankyrin repeat domain 30B pseudogene | -5.158 | 3.769 |
| 249. | **91768** | CABLES1 | Cdk5 and Abl enzyme substrate 1 | -3.493 | 3.709 |
| 250. | **9050** | PSTPIP2 | proline-serine-threonine phosphatase interacting protein 2 | -3.59 | 3.761 |
| 251. | **5366** | PMAIP1 | phorbol-12-myristate-13-acetate-induced protein 1 | -2.356 | 3.204 |
| 252. | **10347** | ABCA7 | ATP binding cassette subfamily A member 7 | -2.586 | 6.988 |
| 253. | **92359** | CRB3 | crumbs cell polarity complex component 3 | -2.723 | 5.89 |
| 254. | **3643** | INSR | insulin receptor | 8.086 | 9.08 |
| 255. | **9294** | S1PR2 | sphingosine-1-phosphate receptor 2 | 2.819 | 4.468 |
| 256. | **100422925** | MIR4322 | microRNA 4322 | 2.613 | 3.3 |
| 257. | **102724908** | LOC102724908 | uncharacterized LOC102724908 | -6.945 | 6.779 |
| 258. | **400685** | LINC01801 | long intergenic non-protein coding RNA 1801 | 4.026 | 3.649 |
| 259. | **115727** | RASGRP4 | RAS guanyl releasing protein 4 | -2.19 | 6.988 |
| 260. | **390937** | ERFL | ETS repressor factor like | 3.314 | 5.013 |
| 261. | **199731** | CADM4 | cell adhesion molecule 4 | -3.864 | 8.062 |
| 262. | **107984140** | LOC107984140 |  | -2.559 | 3.012 |
| 263. | **2512** | FTL | ferritin light chain | -2.233 | 4.984 |
| 264. | **8463** | TEAD2 | TEA domain transcription factor 2 | -2.5 | 3.806 |
| 265. | **22809** | ATF5 | activating transcription factor 5 | -2.189 | 4.458 |
| 266. | **100616483** | MIR4751 | microRNA 4751 | -2.323 | 3.587 |
| 267. | **128853** | DUSP15 | dual specificity phosphatase 15 | -4.506 | 3.248 |
| 268. | **102723442** | LINC01754 | long intergenic non-protein coding RNA 1754 | 3.936 | 4.516 |
| 269. | **5770** | PTPN1 | protein tyrosine phosphatase non-receptor type 1 | -2.182 | 5.146 |
| 270. | **140876** | RIPOR3 | RIPOR family member 3 | -2.943 | 7.011 |
| 271. | **55653** | BCAS4 | breast carcinoma amplified sequence 4 | 2.274 | 12.719 |
| 272. | **10079** | ATP9A | ATPase phospholipid transporting 9A (putative) | -4.242 | 3.517 |
| 273. | **655** | BMP7 | bone morphogenetic protein 7 | 7.9 | 13.069 |
| 274. | **112268270** | LOC112268270 | translation initiation factor IF-2-like | 5.746 | 4.361 |
| 275. | **56937** | PMEPA1 | prostate transmembrane protein, androgen induced 1 | 8.637 | 10.366 |
| 276. | **128611** | ZNF831 | zinc finger protein 831 | 4.179 | 5.134 |
| 277. | **105372832** | LOC105372832 | uncharacterized LOC105372832 | 4.235 | 5.916 |
| 278. | **149992** | ANKRD30BP2 | ankyrin repeat domain 30B pseudogene 2 | -4.349 | 3.296 |
| 279. | **114614** | MIR155HG | MIR155 host gene | -4.222 | 4.62 |
| 280. | **406947** | MIR155 | microRNA 155 | -4.099 | 4.502 |
| 281. | **282566** | LINC00515 | long intergenic non-protein coding RNA 515 | -2.26 | 4.506 |
| 282. | **89766** | UMODL1 | uromodulin like 1 | 4.642 | 3.789 |
| 283. | **107983952** | LOC107983952 | replaced by ID 105372832 | 4.303 | 5.92 |
| 284. | **102725065** | LOC102725065 | uncharacterized LOC102725065 | 3.474 | 3.361 |
| 285. | **649179** | PRAMENP | PRAME N-terminal like, pseudogene | 3.757 | 5.28 |
| 286. | **28771** | IGLV10-67 | immunoglobulin lambda variable 10-67 (pseudogene) | 3.336 | 3.365 |
| 287. | **102724653** | LOC102724653 | uncharacterized LOC102724653 | 4.96 | 4.313 |
| 288. | **7441** | VPREB1 | V-set pre-B cell surrogate light chain 1 | 5.007 | 3.841 |
| 289. | **3543** | IGLL1 | immunoglobulin lambda like polypeptide 1 | 6.967 | 5.612 |
| 290. | **8542** | APOL1 | apolipoprotein L1 | -4.043 | 4.506 |
| 291. | **112268296** | LOC112268296 |  | -3.682 | 4.506 |
| 292. | **3560** | IL2RB | interleukin 2 receptor subunit beta | -5.487 | 3.497 |
| 293. | **55964** | SEPTIN3 | septin 3 | -4.239 | 5.23 |
| 294. | **101927344** | LOC101927344 | uncharacterized LOC101927344 | 4.594 | 3.837 |
| 295. | **112268288** | LOC112268288 | uncharacterized LOC112268288 | -2.573 | 3.311 |
| 296. | **105379413** | LOC105379413 | uncharacterized LOC105379413 | 3.838 | 4.749 |
| 297. | **11040** | PIM2 | Pim-2 proto-oncogene, serine/threonine kinase | -2.554 | 6.362 |
| 298. | **1831** | TSC22D3 | TSC22 domain family member 3 | 2.766 | 4.296 |
| 299. | **2334** | AFF2 | ALF transcription elongation factor 2 | 3.711 | 3.679 |

Supplementary Table 1. 299 dysregulated DEG with the cut-off criteria of fold change> 2 and p ≤ 0.001.
